# A higher burden of metabolic risk factors and underutilization of therapy among women compared to men might influence a poorer prognosis: a study among acute myocardial ifarction patients in Albania, a transitional country in Southeastern Europe

**DOI:** 10.3325/cmj.2015.56.542

**Published:** 2015-12

**Authors:** Sokol Myftiu, Enxhela Sulo, Genc Burazeri, Ilir Sharka, Artan Shkoza, Gerhard Sulo

**Affiliations:** 1 Department of Cardiology, University Hospital “Mother Teresa,” Tirana, Albania; 2 Department of Global Public Health and Primary Care, University of Bergen, Bergen, Norway; 3 School of Public Health, University of Medicine, Tirana, Albania; 4 Department of International Health, School for Public Health and Primary Care, Faculty of Health, Medicine and Life Sciences, Maastricht University, Maastricht, the Netherlands; 5 Department of Biomedical and Experimental Sciences, University of Medicine, Tirana, Albania

## Abstract

**Aim:**

To determine the clinical profile, burden of risk factors, and quality of care among patients hospitalized for an acute myocardial infarction (AMI) with special focus on gender differences.

**Methods:**

The study included 256 AMI patients admitted to the Coronary Care Unit of “Mother Teresa” hospital in Tirana during 2013-2014. We obtained information on patients’ demographic data, AMI characteristics, complications (heart failure [HF] and ventricular fibrillation [VF]), risk factors and medication use prior and during the AMI hospitalization. Age-adjusted Poisson regression analyses were applied to explore gender differences (women vs men) with regard to clinical profile and quality of care and results are expressed as incidence rate ratios (IRR).

**Results:**

55.4% of patients had ≥3 risk factors, 44.5% developed HF, and 5.7% developed VF. Only 40.4% of patients received all 4 medication classes (beta-blockers, angiotensin-converting-enzyme inhibitor/angiotensin receptor blockers, statins, and aspirin) and 46.4% had revascularization. Significantly more women than men were obese, (*P* = 0.042) had diabetes, (*P* = 0.001) developed HF (*P* < 0.001) or experienced a VF episode (*P* < 0.001). After adjusting for age, differences with regard to obesity (IRR = 2.17; 95% confidence interval [CI] 1.15-4.09), diabetes (IRR = 1.35; 95% CI 1.07-1.71), HF (IRR = 1.32; 95% CI 1.02-1.74) and VF (IRR = 2.82; 95% CI 1.07-7.43) remained significant. There were no differences with regard to individual drug classes taken. However, women had fewer revascularization procedures than men (IRR = 0.65; 95% CI 0.43-0.98).

**Conclusion:**

Women were found to have more unfavorable clinical profile, higher complication rates, and underutilization of therapy, which may be influenced by socioeconomic differences between genders and lead to a differential prognosis.

Coronary heart disease (CHD) is recognized as a major cause of mortality, accounting for 1 in 5 deaths in Europe (1). Remarkable achievements in the treatment (2) have led to significant improvements in survival following a coronary event (3-5). Nevertheless, AMI prognosis is still characterized by gender disparities (6) thought to be influenced by differences with regard to age at the first coronary event and comorbidities, but also treatment and secondary prevention.

While during the last two decades many Western countries have experienced declines in CHD mortality and incidence rates, Albania has experienced an increase (7). This was influenced, among other things, by a rapid transition from a totalitarian communist regime to a free, marked-oriented economy, which resulted in a shift from the traditional Mediterranean diet (8) to a “Western” diet, consisting of processed foods richer in sugar, salt, and saturated fats (9).

Despite unfavorable trends in CHD occurrence in Albania, the proportion of gross domestic product allocated to the health care system is lower ( ~ 50%) than in many European countries (10). These limited financial resources are mainly directed toward providing hospitals with sufficient medical supplies and equipment, while epidemiological research and prevention strategies and rehabilitation programs remain underfunded. As a consequence, there is sparse information on coronary patients’ profile, burden of risk factors, disease severity and treatment modalities – all of them important factors influencing AMI prognosis (11,12). Distribution of these factors with regard to age and gender and their clinical significance for prognosis are also not sufficiently investigated. Despite the efforts toward a better integration of women in the economic and social life, gender inequalities still persist, and the woman’s role is often limited to that of a caretaker of her children and the spouse, not leaving her enough time and resources to deal with her own health.

Therefore, we conducted this study to assess the overall burden and potential gender differences in the clinical profile, disease severity, and quality of care among patients hospitalized for an AMI in the Coronary Care Unit (CCU) of ‘Mother Teresa’ hospital in Tirana, Albania.

## Materials and methods

### Study population and data collection

The study included 265 consecutive patients, residents of Tirana admitted during 2013-2014 due to an AMI at the CCU of the University Hospital Center “Mother Teresa.” This is the only public hospital providing residents of Tirana with specialized cardiac care.

Detailed information on age, gender, education, height and weight, systolic and diastolic blood pressure, AMI type (ST-elevation MI [STEMI] vs non ST-elevation MI), location, major complications such as heart failure (HF) and ventricular fibrillation (VF) and treatment was obtained from patients’ medical charts. Glucose and cholesterol level were measured in fasting blood samples. Information on coronary risk factors, (smoking, hypertension, diabetes, dyslipidemia), previous history of AMI, and drug use prior to hospitalization was obtained from patients’ reports.

### Variable definitions

Education was categorized into primary (up to 8 years), secondary (9-12 years), and tertiary (>12 years). Body mass index (BMI) was calculated as weight/height^2^ and patients were categorized as normal (<25), overweight (between 25 and 30), and obese (≥30). According to smoking status patients were categorized into never/former smokers and current smokers. Hypertension was defined as systolic blood pressure (SBP)≥140 mm Hg, diastolic blood pressure (DBP)≥90 mm Hg, or prior use of antihypertensive medications. Diabetic status was categorized as “no diabetes” (glucose levels <5.6 mmol/L), “impaired fasting glucose” (glucose levels between 5.6-6.9 mmol/L), and “diabetes” (glucose levels >6.9 mmol/L). Prior use of hypoglycemic agents (either insulin or oral hypoglycemics) classified the patients into “diabetes” category, regardless of blood glucose levels. Hypercholesterolemia was defined as total cholesterol ≥6.2 mmol/L or prior use of statins. The study protocol was approved by the National Committee for Bio-Medical Ethics in Albania.

### Statistical analyses

Continuous variables are presented as means and standard deviations (SD) whenever their distribution was proved to be normal (Shapiro-Wilk test in STATA). Categorical variables are presented as frequency distributions (absolute numbers and corresponding proportions). Mean values of the continuous variables were compared using independent sample *t* test, while distribution of categorical variables was compared using the χ^2^ test or Fisher exact test in cases of small sample sizes (at least one expected cell <5). Poisson regression models with robust variances were constructed to compare gender differences with regard to clinical profile and quality of care during the AMI hospitalization. These models were adjusted for age and results were expressed as incidence rate ratios (IRR) with 95% confidence intervals (CI) for women vs men (the reference category). Two-sided tests with a 0.05 level of significance were used. Analyses were performed using STATA software (Release 13, StataCorp LP, College Station, TX, USA).

## Results

### Overall analyses

Mean age of participants was 64.9 (12.1) years, and 77.7% were men. There were 38.5% of patients with primary education, 43.8% with secondary, and 17.7% with tertiary education. STEMI was the dominating AMI type (87.1%). In 52.4% of the cases, AMI involved the anterior wall. Only 12.5% of patients had been hospitalized for a previous AMI.

We observed a clustering of risk factors among study participants at the time of hospitalization: 20% of them had 4-5 risk factors, while 55.4% had at least 3 risk factors. Hypertension was observed in 73.6% of patients, followed by smoking (61.5%), hypercholesterolemia (57.5%), diabetes mellitus (DM, 52.8%), and obesity (14.0%).

The proportion of patients with AMI complicated with HF was 44.5%. Of those, 88.1% had impaired left ventricle (LV) function (defined as LV ejection fraction ≤0.45 on echocardiographic examination). An episode of VF during hospitalization was registered in 5.7% of patients (Table 1).

**Table 1:** Characteristics of patients hospitalized for an acute myocardial infarction (AMI)*

Characteristics	Total (n = 265)	Women (n = 59)	Men (n = 206)	*P*
Age (y), mean (standard deviation)	64.9 (12.1)	70.2 (12.7)	63.3 (11.7)	<0.001
Education, n (%):				0.081
primary	102 (38.5)	30 (50.8)	72 (34.9)	
secondary	116 (43.8)	22 (37.3)	94 (45.6)	
tertiary	47 (17.7)	7 (11.9)	40 (19.4)	
AMI characteristics, n (%):				
type (STEMI*)	230 (87.1)	54 (91.5)	176 (85.9)	0.337
location:				0.448
anterior	139 (52.4)	31 (51.4)	108 (52.6)	
inferior	106 (40.1)	26 (45.7)	80 (38.8)	
lateral	20 (7.5)	2 (2.9)	18 (8.5)	
previous AMI	33 (12.5)	8 (13.6)	25 (12.1)	0.723
Risk factor, n (%):				
obesity	37 (14.0)	13 (22.0)	24 (11.7)	0.041
hypercholesterolemia	153 (57.7)	33 (55.9)	120 (58.3)	0.751
diabetes	140 (52.8)	40 (67.8)	100 (48.5)	0.010
hypertension	195 (73.6)	47 (79.7)	148 (71.8)	0.230
smoking (current)	163 (61.5)	10 (17.0)	153 (74.3)	<0.001
Number of risk factors, n (%):				
≥1	261 (98.5)	58 (98.3)	203 (98.5)	0.747
≥2	224 (84.5)	49 (83.0)	175 (84.9)	0.638
≥3	147 (55.4)	28 (47.5)	119 (57.8)	0.160
4-5	54 (20.3)	8 (13.5)	46 (22.3)	0.142
AMI complication, n (%):				
heart failure	118 (44.5)	36 (61.0)	82 (39.8)	<0.001
impaired LV function	105 (39.6)	33 (55.9)	72 (35.0)	<0.001
ventricular fibrillation	15 (5.7)	7 (11.9)	8 (3.9)	<0.001
any complication	129 (48.7)	37 (62.7)	92 (44.7)	<0.001

*STEMI – ST-elevation myocardial infarction; LV – left ventricle.

The utilization rates were higher for statins (96.6%) and aspirin (94.7%) compared to beta-blockers (55.5%) or angiotensin-converting enzyme inhibitors (ACEI)/angiotensin receptor blockers (ARB) (61.5%). Only 40.4% of patients received 4 drug classes, followed by 30.6% receiving 3, 26.0% receiving 2, and 3.0% receiving only 1 class. A total of 46.4% of patients had revascularization; 44.5% underwent percutaneous coronary intervention or coronary artery bypass grafting, and only 3.8% had thrombolysis (Table 2).

**Table 2:** Gender-specific treatment during hospitalization for an acute myocardial infarction (AMI)

	No. (%) of patients			
Treatment	total (n = 265)	women (n = 59)	men (n = 206)	*P*
Drug class:				
beta-blockers	147 (55.5)	27 (45.8)	120 (58.3)	0.089
ACEI/ARB	163 (61.5)	33 (55.9)	130 (63.1)	0.318
statins	256 (96.6)	56 (94.9)	200 (97.1)	0.417
aspirin	251 (94.7)	52 (88.1)	199 (96.6)	0.010
Number of drug classes:				
4	107 (40.4)	17 (28.8)	90 (43.7)	<0.001
3	81 (30.6)	18 (30.5)	63 (30.6)	0.991
2	69 (26.0)	22 (37.3)	47 (22.8)	0.026
1	8 (3.0)	2 (3.4)	6 (2.9)	0.932
Revascularization:				
PCI or CABG	118 (44.5)	15 (25.4)	103 (50.0)	<0.001
any type^†^	123 (46.4)	18 (30.5)	105 (51.0)	<0.001
PCI	77 (29.2)	9 (15.3)	68 (33.2)	<0.001
CABG	42 (15.9)	6 (10.3)	36 (17.6)	<0.001
thrombolysis	10 (3.8)	3 (5.1)	7 (3.4)	0.453

*ACEI – angiotensin-converting enzyme inhibitor; ARB – angiotensin receptor blocker; PCI – percutaneous coronary intervention; CABG – coronary artery bypass grafting.

^†^PCI, CABG or thrombolysis.

### Gender-specific analyses

Women were on average 7 years older than men (70.2 vs 63.3 years; *P* < 0.001). No significant gender differences were found with regard to education (*P* = 0.081), AMI type (*P* = 0.337), localization (*P* = 0.448), or history of AMI (*P* = 0.723). A higher proportion of women were obese (*P* = 0.041) and had diabetes (*P* = 0.010), whereas men more frequently smoked (*P* < 0.001). The clustering of risk factors was similar between men and women (Table 1). A higher proportion of female patients developed HF (*P* < 0.001), VF (*P* < 0.001), or both (*P* < 0.001) during hospital stay (Table 1).

**Figure 1:**
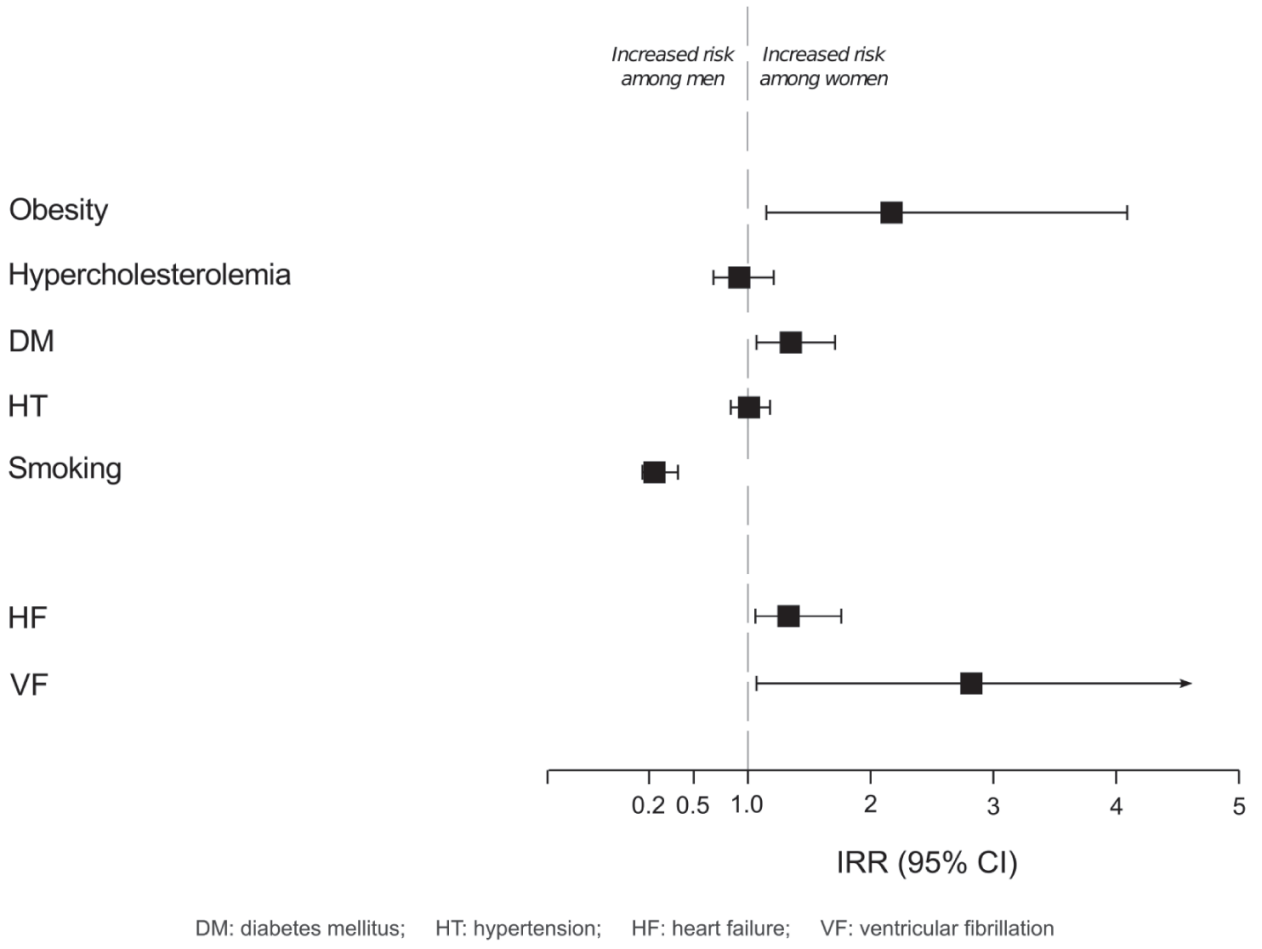
Gender differences in coronary risk factors and complications among patients hospitalized with an acute myocardial infarction

When adjusting for age, gender differences with regard to obesity (IRR = 2.17; 95% CI 1.15-4.09), diabetes (IRR = 1.35; 95% CI 1.07-1.71), and smoking (IRR = 0.24; 95% CI 0.14-0.43) remained significant. Conversely, no significant differences were observed with regard to hypertension and hypercholesterolemia (Figure 1, Supplementary Table 1(web extra table 1)). Women had a higher risk of developing HF (IRR = 1.32; 95% CI 1.02-1.74) or VF (IRR = 2.82; 95% CI 1.07-7.43) than men (Figure 1 and Table 1, Supplementary Table 1(web extra table 1)).

Women less often received beta-blockers, ACEI/ARBs, and statins, though such differences were not significant. Only the proportion of women receiving aspirin was significantly lower (*P* = 0.010). Fewer women received all 4 drug classes (*P* < 0.01) or had revascularization procedures (*P* < 0.001) (Table 2). Analyses stratified by presence of HF suggested that the presence of gender differences with regard to treatment went beyond the severity of AMI (Table 3).

**Table 3:** Gender-specific treatment during hospitalization for an acute myocardial infarction (AMI) by presence of heart failure (HF)

Treatment	No. (%) of patients without HF (n = 147)		No. (%) of patients with HF (n = 108)	
	women (n = 23)	men (n = 124)	women (n = 36)	men (n = 82)
Drug class:				
beta-blockers	15 (65.2)	83 (66.9)	12 (33.3)	37 (45.1)
ACEI/ARB	15 (65.2)	88 (71.0)	18 (50.0)	42 (51.2)
aspirin	19 (82.6)	120 (96.8)	33 (91.7)	79 (96.3)
statins	22 (95.7)	122 (98.4)	34 (94.4)	78 (95.1)
Number of drug classes:				
4	9 (39.1)	63 (50.8)	8 (22.2)	27 (32.9)
3	8 (34.8)	41 (33.1)	10 (27.8)	22 (26.8)
2	5 (21.7)	18 (14.5)	17 (47.2)	29 (35.4)
1	1 (4.4)	2 (1.6)	1 (2.8)	4 (4.9)
Revascularization:				
PCI or CABG	7 (30.4)	65 (52.4)	8 (22.2)	38 (46.3)
any type^†^	8 (34.7)	67 (54.0)	10 (27.7)	38 (46.3)

*ACEI – angiotensin-converting enzyme inhibitor; ARB – angiotensin receptor blocker; PCI – percutaneous coronary intervention; CABG – coronary artery bypass grafting.

†PCI, CABG, or thrombolysis.

**Figure 2:**
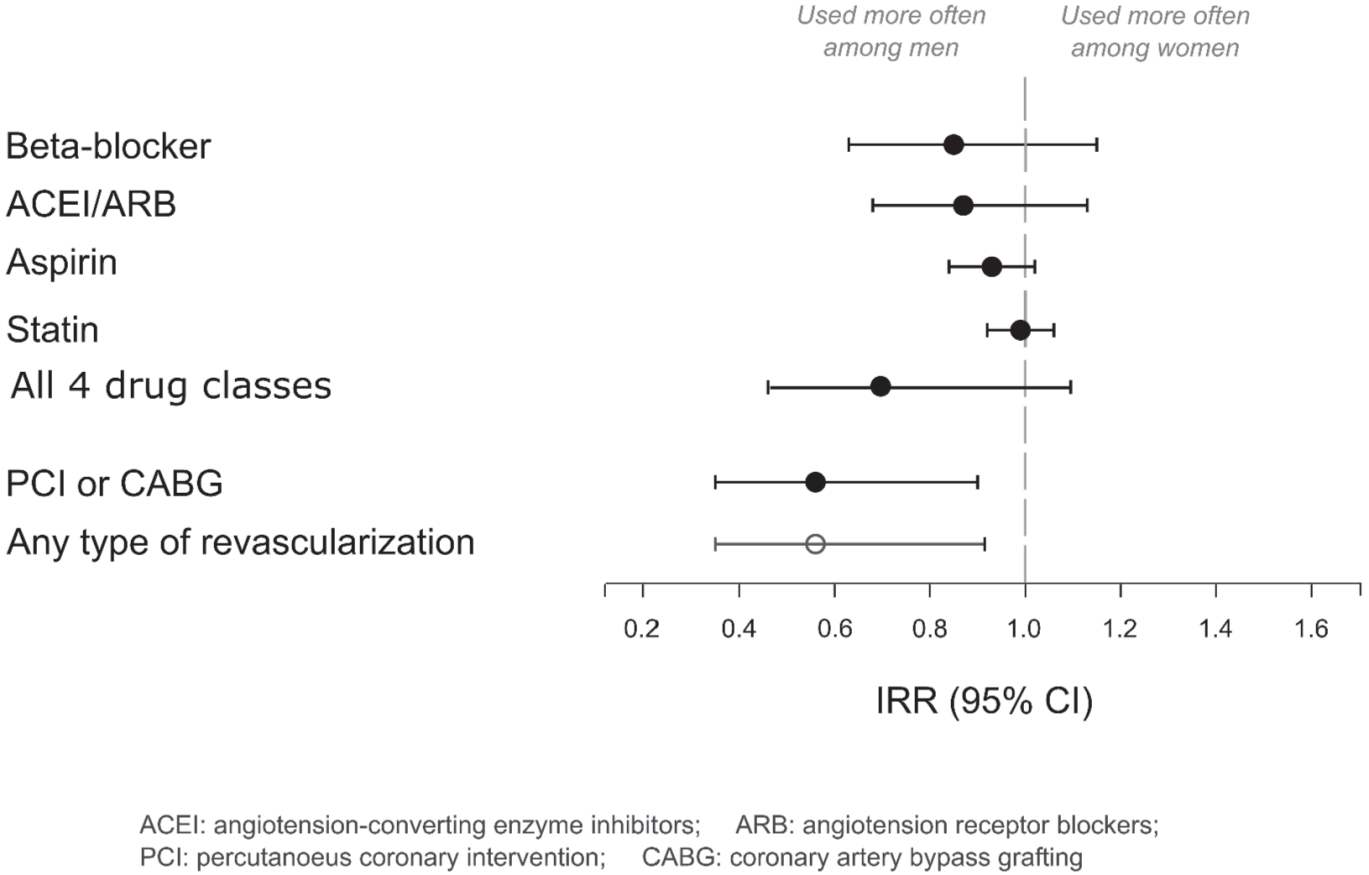
Gender differences in receiving evidence-based therapy among patients hospitalized for an acute myocardial infarction (AMI)

After adjusting for age, the uptake of each individual drug class as well as of all 4 drug classes was lower among women, although differences were not significant. Women less often had revascularization: either of any type (IRR = 0.65; 95% CI 0.43-0.98) or invasive (IRR = 0.56; 95% CI 0.35-0.89) (Figure 2, Supplementary Table 2(web extra table 2)). The results did not change when the severity of AMI was taken into account (Supplementary Table 2(web extra table 2)).

## Discussion

We observed a clustering (≥3) of coronary risk factors in more than half of our study population. AMI was complicated with HF or VF in 48.7% of the cases, demonstrating a severe clinical expression of the disease. Only 40.4% of patients received 4 classes of medications (beta-blockers, ACEI/ARB, statins, and aspirin) and 46.4% had revascularization. Our study population was also characterized by gender differences with regard to clinical profile, disease severity, and quality of care. Women were more often presenting a more severe form of the disease and received less often optimal therapy compared to men. Such differences in the treatment were independent from age and clinical severity.

Information on the risk factors’ burden among coronary patients in Albania and their treatment is missing. So far only one study has shown that among 467 consecutive patients hospitalized for incident AMI or unstable angina pectoris, the proportion of obesity, hypertension, and diabetes was higher among women (13). These findings are in line with those presented in this work, despite some differences that could be attributable to older age and higher risk profile of our study population.

Similar to other international studies (14,15), women in our study were at higher risk of developing HF than men, even after correcting for age at the hospitalization. Previous studies have shown a longer delay in seeking medical assistance among women than men due to less specific symptoms and a lower awareness of potential risk of coronary event (16). There is also evidence that women’s perception and responsive actions upon symptoms onset are different from those observed among men (17). These factors increase the time of myocardial exposure to ischemia among women and thus the risk of developing HF. The risk of HF among women might be mediated through the direct role of obesity and diabetes, known to be strong, independent predictors of HF (18,19). Lastly, our findings can also be explained by a lower socioeconomic status of women in Albanian society (20) and influence of patriarchy (21). These factors may lead to women’s inability to understand and hence promptly react to symptoms, causing delays in seeking medical assistance.

We observed that fewer women than men received optimal treatment as recommended by AMI treatment guidelines. Similar to a previous study conducted in Canada (22), we observed that these gender differences went beyond the effect of age and AMI severity, suggesting the potential involvement of other factors.

Underutilization of revascularization procedures among women compared to men could, to a certain extent, be explained by differences in disease expression. Women less often present with obstructive coronary artery disease (23-25) and in their case microvascular disease is the underlying mechanism causing adverse events (including AMI). The etiology of AMI among women involves plaque disruption, rupture and ulceration with distal embolization, and/or vasospasm, while invasive diagnostic and treatment approaches are more appropriate to treat the “male phenotype”-CHD (ie, obstructive atherosclerosis of the epicardial coronary arteries) (26).

This is the first analysis providing information on patients’ profile, quality of care, and gender differences among AMI patients in Albania, a particularly under-researched population that is still undergoing a rapid transition characterized by lifestyle changes and chronic disease patterns. The information obtained in our study highlights the need for efficient, gender-specific primary and secondary prevention measures. Our results demonstrate that the absence of prevention interventions in the general population not only increases the number of AMI cases but also deteriorates their clinical profile. This renders the treatment more challenging and patients’ prognosis poorer.

Nonetheless, our study may have several limitations. Due to a relatively small sample size, we were unable to detect potential differences related to individual medication classes. However, our findings are in line with studies including a much larger number of participants. We did not have information on the absolute or relative contraindications to individual medication classes. Gender differences in the quality of care may have been related to differences in the health profile (ie, co-existing medical conditions representing contraindications to AMI treatment) between genders. However, such information was not available in our study and, hence, this hypothesis could not be tested. On the face of it though, there is no plausible reason to assume that there are gender differences in terms of quality of care related to different health profiles between men and women in Albania. In our study the category of “never/former” smokers included participants who did not smoke at least one year prior to the hospitalization. No detailed information on time since the last cigarette was available for those who smoked previously but stopped at some point. We regard this as another limitation of this study as time from quitting smoking is inversely associated with the risk for CHD (27). We also did not have the information on the delays from symptom onset to arrival at the hospital and thus were unable to distinguish between patients presenting with signs of HF on admission or those developing HF during the hospital stay.

The presence of many coronary risk factors and AMI clinical severity, combined with a suboptimal coronary care (especially among women) can adversely influence the prognosis of coronary patients in Albania, especially when considering that the country lacks structured rehabilitation programs and standardized secondary prevention interventions. Further studies are needed to elucidate the factors influencing the clinical profile of Albanian patients and suboptimal quality of coronary care.

## References

[1] Nichols M, Townsend N, Scarborough P, Rayner M (2013). Cardiovascular disease in Europe: epidemiological update.. Eur Heart J.

[2] Nabel EG, Braunwald E (2012). A tale of coronary artery disease and myocardial infarction.. N Engl J Med.

[3] Sulo E, Vollset SE, Nygard O, Sulo G, Igland J, Egeland GM (2015). Trends in 28-day and 1-year mortality rates in patients hospitalized for a first acute myocardial infarction in Norway during 2001-2009: a “Cardiovascular disease in Norway” (CVDNOR) project.. J Intern Med.

[4] Koopman C, Bots ML, van Oeffelen AA, van Dis I, Verschuren WM, Engelfriet PM (2013). Population trends and inequalities in incidence and short-term outcome of acute myocardial infarction between 1998 and 2007.. Int J Cardiol.

[5] Kostis WJ, Deng Y, Pantazopoulos JS, Moreyra AE, Kostis JB (2010). Myocardial Infarction Data Acquisition System Study G. Trends in mortality of acute myocardial infarction after discharge from the hospital.. Circ Cardiovasc Qual Outcomes.

[6] Bucholz EM, Butala NM, Rathore SS, Dreyer RP, Lansky AJ, Krumholz HM (2014). Sex differences in long-term mortality after myocardial infarction: a systematic review.. Circulation.

[7] Nichols M, Townsend N, Scarborough P, Rayner M (2014). Cardiovascular disease in Europe 2014: epidemiological update.. Eur Heart J.

[8] Gjonca A, Bobak M (1997). Albanian paradox, another example of protective effect of Mediterranean lifestyle?. Lancet.

[9] Mone I, Bulo A (2012). Total fats, saturated Fatty acids, processed foods and acute coronary syndrome in transitional Albania.. Mater Sociomed..

[10] Bank TW. Health expenditure, total (% of GDP) 2015. Available from: http://data.worldbank.org/indicator/SH.XPD.TOTL.ZS/countries/AL-AT?display=default*.* Accessed: December 4, 2015.

[11] Bruthans J, Cifkova R, Lanska V, O'Flaherty M, Critchley JA, Holub J (2014). Explaining the decline in coronary heart disease mortality in the Czech Republic between 1985 and 2007.. Eur J Prev Cardiol..

[12] Hughes J, Kee F, O'Flaherty M, Critchley J, Cupples M, Capewell S (2013). Modelling coronary heart disease mortality in Northern Ireland between 1987 and 2007: broader lessons for prevention.. Eur J Prev Cardiol..

[13] Burazeri G, Goda A, Sulo G, Stefa J, Roshi E, Kark JD (2007). Conventional risk factors and acute coronary syndrome during a period of socioeconomic transition: population-based case-control study in Tirana, Albania.. Croat Med J.

[14] Hung J, Teng TH, Finn J, Knuiman M, Briffa T, Stewart S (2013). Trends from 1996 to 2007 in incidence and mortality outcomes of heart failure after acute myocardial infarction: a population-based study of 20,812 patients with first acute myocardial infarction in Western Australia.. J Am Heart Assoc..

[15] Shah RV, Holmes D, Anderson M, Wang TY, Kontos MC, Wiviott SD (2012). Risk of heart failure complication during hospitalization for acute myocardial infarction in a contemporary population: insights from the National Cardiovascular Data ACTION Registry.. Circ Heart Fail.

[16] Nguyen HL, Saczynski JS, Gore JM, Goldberg RJ (2010). Age and sex differences in duration of prehospital delay in patients with acute myocardial infarction: a systematic review.. Circ Cardiovasc Qual Outcomes.

[17] Lovlien M, Schei B, Hole T (2007). Prehospital delay, contributing aspects and responses to symptoms among Norwegian women and men with first time acute myocardial infarction.. Eur J Cardiovasc Nurs.

[18] Horwich TB, Fonarow GC (2010). Glucose, obesity, metabolic syndrome, and diabetes relevance to incidence of heart failure.. J Am Coll Cardiol.

[19] Nicklas BJ, Cesari M, Penninx BW, Kritchevsky SB, Ding J, Newman A (2006). Abdominal obesity is an independent risk factor for chronic heart failure in older people.. J Am Geriatr Soc.

[20] Health IoP. National Health Report 2014. Available from: http://ishp.gov.al/raport-shendetesor-kombetar/*.* Accessed: December 4, 2015.

[21] Burazeri G, Roshi E, Jewkes R, Jordan S, Bjegovic V, Laaser U (2005). Factors associated with spousal physical violence in Albania: cross sectional study.. BMJ.

[22] Bugiardini R, Yan AT, Yan RT, Fitchett D, Langer A, Manfrini O (2011). Factors influencing underutilization of evidence-based therapies in women.. Eur Heart J.

[23] Heer T, Schiele R, Schneider S, Gitt AK, Wienbergen H, Gottwik M (2002). Gender differences in acute myocardial infarction in the era of reperfusion (the MITRA registry).. Am J Cardiol.

[24] Berger JS, Elliott L, Gallup D, Roe M, Granger CB, Armstrong PW (2009). Sex differences in mortality following acute coronary syndromes.. JAMA.

[25] Rosengren A, Wallentin L, Gitt AK, Behar S, Battler A, Hasdai D (2004). Sex, age, and clinical presentation of acute coronary syndromes.. Eur Heart J.

[26] Wenger NK (2012). Women and coronary heart disease: a century after Herrick: understudied, underdiagnosed, and undertreated.. Circulation.

[27] Kawachi I, Colditz GA, Stampfer MJ, Willett WC, Manson JE, Rosner B (1994). Smoking cessation and time course of decreased risks of coronary heart disease in middle-aged women.. Arch Intern Med.

