# Supplementary material for: A higher burden of metabolic risk factors and underutilization of therapy among women compared to men might influence a poorer prognosis: a study among acute myocardial ifarction patients in Albania, a transitional country in Southeastern Europe
Source: Croat Med J. 2015 Dec;56(6):542–9. doi: 10.3325/cmj.2015.56.542 (PMC4707925; doi:10.3325/cmj.2015.56.542)
Supplement: Supplementary Table 1 [file CroatMedJ_56_s004.pdf]

Online supplemental material, Table 1. Gender differences in coronary risk factors, and AMI complications among patients hospitalized for an acute myocardial infarction (AMI)

	IRR (95% CI)	
	Model 1	Model 2
Risk factor		
Obesity	1.89 (1.03 - 3.48)	2.17 (1.15 - 4.09)
Hypercholesterolemia	0.96 (0.74 - 1.24)	0.93 (0.72 - 1.21)
Diabetes	1.40 (1.11 - 1.75)	1.35 (1.07 - 1.71)
Hypertension	1.11 (0.95 - 1.29)	1.01 (0.86 - 1.18)
Smoking	0.23 (0.13 - 0.40)	0.24 (0.14 - 0.43)
AMI complication		
Heart failure	1.53 (1.18 - 2.00)	1.32 (1.02 - 1.74)
Impaired LV function	1.62 (1.21 - 2.18)	1.44 (1.06 - 1.96)
Ventricular fibrillation	3.06 (1.15 - 8.09)	2.82 (1.07 - 7.43)

IRR: incidence rate ratio obtained from Poisson regression analyses, comparing women to men

LV: left ventricle

Model 1: Unadjusted

Model 2: Adjusted for age
