# Supplementary material for: A higher burden of metabolic risk factors and underutilization of therapy among women compared to men might influence a poorer prognosis: a study among acute myocardial ifarction patients in Albania, a transitional country in Southeastern Europe
Source: Croat Med J. 2015 Dec;56(6):542–9. doi: 10.3325/cmj.2015.56.542 (PMC4707925; doi:10.3325/cmj.2015.56.542)
Supplement: Supplementary Table 2 [file CroatMedJ_56_s005.pdf]

Online supplemental material, Table 2. Gender differences in receiving evidence-based therapy among patients hospitalized for an acute myocardial infarction (AMI)

	IRR (95% CI)		
	Model 1	Model 2	Model 3
Beta-blocker	0.79 (0.58 - 1.06)	0.85 (0.63 - 1.15)	-
ACEI/ARB	0.89 (0.69 - 1.14)	0.87 (0.68 - 1.13)	-
Aspirin	0.91 (0.83 - 1.01)	0.93 (0.84 - 1.02)	-
Statin	0.98 (0.92 - 1.04)	0.99 (0.92 - 1.06)	-
All 4 drug classes	0.66 (0.43 – 1.01)	0.69 (0.45 – 1.08)	
Revascularization			
PCI or CABG	0.51 (0.32 - 0.80)	0.56 (0.35 - 0.89)	0.56 (0.36 - 0.90)
Any type of revascularization*	0.60 (0.41 - 0.90)	0.65 (0.43 - 0.98)	0.64 (0.42 - 0.98)

IRR: incidence rate ratio obtained from Poisson regression analyses, comparing women to men

ACEI: angiotensin-converting enzyme inhibitor

ARB: angiotensin receptor blocker

PCI: percutaneous coronary intervention

CABG: coronary bypass surgery

Model 1: Unadjusted
Model 2: Adjusted for age
Model 3: adjusted for age and AMI complications
*   PCI, CABG or thrombolysis
